# Effect of intergenerational exchange patterns and intergenerational relationship quality on depressive symptoms in the elderly: An empirical study on CHARLS data

**DOI:** 10.3389/fpubh.2022.1009781

**Published:** 2022-10-03

**Authors:** Ranran Zheng, Mingyang Yu, Li Huang, Fang Wang, Baizhi Gao, Duanduan Fu, Jinghui Zhu, Guilin Liu

**Affiliations:** ^1^School of Public Health and Management, Wenzhou Medical University, Wenzhou, China; ^2^Integrated Office of Party and Government, First Affiliated Hospital Zhejiang Chinese Medicine University, Hangzhou, China

**Keywords:** intergenerational exchange pattern, intergenerational relationship quality, active aging, the elderly, depressive symptoms

## Abstract

**Background:**

As the population ages with fewer children, depression symptoms are increasing among the elderly who lack companionship. Intergenerational support is closely related to depression in the elderly; hence how the behavioral patterns and emotional quality of intergenerational support affect depressive symptoms in the elderly should be further explored.

**Objective:**

To study the effects of intergenerational exchange patterns and intergenerational relationship quality on depressive symptoms in the elderly.

**Methods:**

A total of 8,015 people over 60 years old in CHARLS in 2018 were selected as the object of this study. First, the correlation between demographics, economic conditions, health status, intergenerational support patterns, intergenerational relationship quality, and depressive symptoms in the elderly were analyzed. Three regression analysis models were established to analyze the relationship between control variables, intergenerational support patterns, intergenerational relationship quality, and depressive symptoms in the elderly. Results: Among the intergenerational economic, care, and emotional exchange modes, the risk of depressive symptoms in the elderly in the mutual support group was 31.8, 38.4, and 25.5% lower than that in the non-communication group. Compared with the elderly with very poor intergenerational relationship quality, the elderly with good, very good, and excellent intergenerational relationship quality had 74.5, 84.0, and 85.6% lower risk of depressive symptoms.

**Discussion:**

Different cultural backgrounds also affect intergenerational exchange patterns and depression in the elderly. During the study of depressive symptoms, two aspects relating to intergenerational support should be considered behaviorally and emotionally: the intergenerational exchange model and the intergenerational relationship quality. As depression in the elderly is affected by multiple factors, the participation and joint efforts of the whole society are required to reduce depressive symptoms in the elderly and realize active aging.

**Conclusion:**

The intergenerational exchange pattern of mutual support and the higher quality of the intergenerational relationship can significantly reduce the depressive symptoms of the elderly.

## Introduction

About 20% of people over 60 years old have varying degrees of depression worldwide (1). They have a significantly reduced quality of life, and severe ones have suicidal tendencies (2, 3). Among those who have committed suicide, 50–70% have suffered from depression (4). Depression and suicide in older adults have garnered much attention because of their vulnerability (5). China has entered an aging society, with the proportion of the elderly in the total population rising to 18.7% (6). According to the World Health Organization (WHO), the prevalence of depression among people aged 65 years and above exceeded 10% in 2018, suggesting that depression has become a significant disease that impairs the health of the elderly (7). The outbreak and continuous spread of Covid-19 in recent years have significantly challenged the mental health of the elderly (8, 9). How to release the depression of the elderly and achieve active aging has become a global social problem (10, 11).

Studies show that intergenerational support is closely related to depressive symptoms in the elderly (12–14). Intergenerational support refers to the economic, care, and emotional interactions between parents and their children within a family, including intergenerational resource exchange and intergenerational relationship quality.

### Intergenerational support patterns

According to the different combinations of the content and direction of intergenerational resource exchange, an intergenerational exchange can be divided into different patterns. Studies on the relationship between intergenerational support and elderly depressive symptoms mainly concentrate on two aspects. The first is the study on the relationship between the content of intergenerational resource exchange and depressive symptoms in the elderly. In terms of economic support, Marie et al. (15) showed that intergenerational economic support could meet the material needs of the elderly, thus increasing their life satisfaction by 14.4%. However, according to the research by Fingerman (16), excessive intergenerational economic support could damage the self-esteem of the elderly, making them feel powerless and thus damaging their mental health. Tang et al. (17) found that intergenerational care support could enable the elderly to get good care, alleviate diseases, and help maintain good health. Other studies have shown that the influence of intergenerational care support on the mental health of the elderly is not insignificant (18) and even harmful (19). In the field of intergenerational emotional support, Roh et al. (20) showed that compared with the above two factors, emotional support could more effectively maintain the mental health of the elderly and reduce the prevalence of depression. However, this effect was not as apparent for excessive or insufficient emotional support, according to research by Teixeira et al. (21). The second is the study on the relationship between the intergenerational support direction and depressive symptoms in the elderly. The intergenerational support direction can be divided into forwarding (parents provide support for children), backward (children provide support for parents), and mutual (children and parents provide support for each other) support. Bonsang et al. (22) found that care support from children hurt fathers' self-rated health and economic support for mothers' self-rated health. Guoping et al. (23) found that intergenerational support from the elderly with poor economic conditions in rural areas could increase children's life stress and the risk of depression in them. Abolfathi et al. (24) found that the elderly who could give feedback to their children for receiving intergenerational support was less depressed because they felt that they were “important” and “useful”.

### Intergenerational relationship quality

Essentially, intergenerational support is a kind of purposeful interaction between generations. Beyond the essential characteristics of the two interacting parties, the expression form and result of interactive behavior in real life are also affected by the subjective emotion factor of both parties: the quality of intergenerational relationships. This is a critical factor to investigate (25). Most scholars measure it with such objective indicators as the intensity and frequency of intergenerational support. Huang et al. (26) measured the intergenerational relationship by using the intensity of intergenerational support, finding that older people who interacted with their children more closely were mentally healthier. Teixeira et al. (21) discovered a link between the frequency of intergenerational support and the mental health of the elderly. Yang et al. (27), using children's subjective evaluation to measure the intergenerational relationship, found that the intergenerational relationship had effects on the health and well-being of both generations. The content, direction, and quality of intergenerational support had specific effects on the level of depression in the elderly, but no uniform conclusion has been reached.

### The current study

There are two limitations to current studies. First, most studies only analyze the relationship between the content or direction of intergenerational resource exchange and the depressive symptoms in the elderly separately, with few focusing on the combination of the content and direction. Second, both the content and direction of intergenerational support are closely related to depressive symptoms in the elderly. The results will inevitably be biased if we only study one of them. While there are few studies on the relationship between intergenerational relationship quality and depressive symptoms in the elderly, objective indicators such as the intensity and frequency of intergenerational support are often used as alternative variables of intergenerational relationship quality, which is indirect speculation that is not very accurate. Intergenerational relationship quality is more of a subjective feeling, and the elderly's subjective evaluation of the intergenerational relationship can better reflect its intergenerational relationship.

The innovations of this study mainly include two aspects. First, the content and direction of intergenerational support are integrated and conceptualized as intergenerational exchange patterns to comprehensively study the relationship between different intergenerational exchange patterns and depressive symptoms in the elderly. Second, the elderly's subjective evaluation of the intergenerational relationship is taken as an indicator to measure the intergenerational relationship quality, and the relationship between the intergenerational relationship quality and intergenerational exchange patterns and the depressive symptoms in the elderly is investigated.

## Methods

### Participants

Data for this study were obtained from the China Health and Retirement Longitudinal Study (CHARLS) in 2018. The CHARLS began in 2008 and was followed up every 2–3 years, with samples collected through stratified random sampling from 150 county-level and 450 village-level units nationwide. In 2018, 19,744 samples were obtained from CHARLS. Respondents aged 60 years and above as of December 31, 2018, were selected as participants of this study, from which those with missing data were eliminated, and finally, 8,015 valid samples were obtained. All the respondents signed informed consent at the time of participation, and this study was approved by the Institutional Review Board of Peking University (IRB00001052-11014).

### Measures

#### Depressive symptoms

In this paper, whether the elderly have depressive symptoms was regarded as the dependent variable. In the CHARLS, Andresen's 1994 revision of the Center for Epidemiologic Studies Depression Scale of the 10-item short table (CES-D-10) was used to measure the degree of depression in respondents. The scale consists of ten questions with four answer options assigned to 0, 1, 2, and 3, from positive to negative, respectively. Respondents were asked to rate the ten questions based on their feelings and behaviors in the previous week, and the total score was the final score, ranging from 0 to 30. The questionnaire is listed in Table 1. Those with a final score ≥10 were determined to have depressive symptoms (28), and the answer was assigned 1; those with a final score < 10 were determined to have no depressive symptoms, and the answer was assigned 0. The study showed that the CES-D-10 scale has sufficient reliability and validity (29). Besides, studies based on CHARLS have confirmed that the CES-D-10 can effectively measure the depression level of the elderly population in China (30). With a short response time and high recovery rate, the scale has greater application potential in ample survey research. However, it is primarily used to assess the severity of depressive symptoms rather than as a diagnostic tool.

**Table 1:** CES-D-10 Scale items.

**Scale**	**Items**
CES-D-10	(1)I was bothered by things that don't usually bother me.
	(2)I had trouble keeping my mind on what I was doing.
	(3)I felt depressed.
	(4)I felt everything I did was an effort.
	(5)I felt hopeful about the future.
	(6)I felt fearful.
	(7) My sleep was restless.
	(8)I was happy.
	(9)I felt lonely.
	(10)I could not get ”going”.

#### Intergenerational support patterns

In this study, three types of intergenerational support were explored: the economic ties between participants and their children (economic support), mutual care between participants and their children (care support), and regular meetings or contact between participants and their children (emotional support). To measure whether the elderly received or provided intergenerational support, participants were asked questions about it. See Table 2 for specific questionnaires.

**Table 2:** Questionnaire of intergenerational support.

**Scale**	**Items**	**Response options**	**Categorization**
Intergenerational economic support	(1) During last year, what was the amount of economic support received from [Child Name]?	>0	Receive
		< 0	No receive
	(2) During last year, what was the amount of economic support provided to [Child Name]?	>0	Provide
		< 0	No Provide
Intergenerational care support	(3) Who most often helps you with (dressing, bathing, eating, getting out of bed, using the toilet, controlling urination and defecation, doing chores, preparing hot meals, shopping, managing money, making phone calls, taking medications)?	Children	Receive
	(4) Suppose that in the future, you needed help with basic daily activities like eating or dressing. Do you have relatives or friends (besides your spouse/partner) who would be willing and able to help you over a long period of time?	Other options	No receive
	(5) During last year, did you/your spouse spend time in taking care of your grandchildren?	Yes	Provide
		No	No Provide
Intergenerational emotional support	(6) How often do you contact with [Child Name] on phone/by message/ on WeChat/ by mail/ by email?	Almost never	No receive
		Other options	Receive
	(7) How often do you contact with [Child Name] on phone/by message/ on WeChat/ by mail/ by email?	Almost never	No Provide
		Other options	Provide

Based on the responses of receiving and providing intergenerational support, the exchange modes were divided into four types: no exchange, only provide, only receive, and mutual support. The first type of “no exchange” means that the respondent has neither provided nor received any support for a year. The second “only receive” means that the respondent only received support without providing any support. The third “Only provide” means that the respondent only provided support without receiving any support for 1 year. The last “mutual support” means that the respondent received and provided support simultaneously. Because effective support is bidirectional, there are two types of intergenerational emotion exchange modes: no exchange and mutual support.

#### Intergenerational relationship quality

The subjective evaluation of the respondents was used in this paper to measure the quality of intergenerational relationships. CHARLS asked respondents to answer, “Are you satisfied with your relationship with your children?”, and the answers were “extremely satisfied,” “very satisfied,” “relatively satisfied,” “not very satisfied,” and “very dissatisfied.” These answers represented five grades of intergenerational relationship quality: “excellent,” “very good,” “good,” “not good,” and “extremely poor”.

#### Demographic variables

Other control factors, including demographics, economic conditions, and health status, also affect the presence of depressive symptoms in the elderly (31, 32). In this paper, the three types of control variables were introduced, among which demographic variables included age, gender, and marital status; economic condition variables included working status, education, residence, and medical insurance status; and health status variables included the presence of chronic diseases and disabilities.

### Analytic strategies

First, CHARLS database indicators were screened, grouped, and assigned. Variables and assignments are shown in Table 3. Second, a descriptive analysis of the participants' variables was carried out, including their demographics, financial and health status, intergenerational exchange patterns, and the quality of their intergenerational relationships. Third, a one-way ANOVA was conducted to determine whether there was a significant correlation between control variables, intergenerational support patterns, intergenerational relationship quality, and depressive symptoms in the elderly. Fourth, three binary logistic regression models were established to study the effects of control variables, intergenerational support patterns, and intergenerational relationship quality on depressive symptoms in the elderly.

**Table 3:** Variables and assignments.

	**Variable**	**Assignment**
Dependent variable	Depressive symptoms	0 = No depressive symptoms, 1 = With depressive symptoms
Independent variables	Intergenerational economic exchange pattern	1 = No exchange, 2 = Only provide, 3 = Only receive, 4 = Mutual support
	Intergenerational care exchange pattern	1 = No exchange, 2 = Only provide, 3 = Only receive, 4 = Mutual support
	Intergenerational emotional exchange pattern	0 = No exchange, 1 = Mutual support
	Intergenerational relationship quality	1 = Extremely poor, 2 = Not good, 3 = Good, 4 = Very good, 5 = Excellent
Control variables	Gender	0 = Male, 1 = Female
	Age	1 = 60–69 years, 2 = 70–79 years, 3 = over 80 years
	Marital status	0 = Unmarried, 1 = Married
	Working status	0 = Non-working, 1 = Working
	Education	1 = Illiteracy, 2 = Primary school, 3 = Junior high school, 4 = Senior high school and above
	Type of medical insurance	1 = No insurance, 2 = Medical insurance for urban workers, 3 = Medical insurance for urban and rural residents, 4 = Medical insurance for urban residents, 5 = New rural cooperative medical insurance, 6 = Other
	Living environment	0 = Rural, 1 = Urban
	Disability status	0 = No, 1 = Yes
	Chronic diseases	0 = No, 1 = Yes

## Results

### Demographic characteristics

Table 4 describes the investigation samples. Depressive symptoms were found in 37.8% of the sample. The quality of intergenerational relationships was relatively good (39.1%) or very good (49.1%). In terms of the mode of intergenerational exchange, more than half (54%) of the elderly said they had no intergenerational economic exchange with their children. In the intergenerational care exchange mode, the elderly in the only receiving group accounted for the largest proportion (45.7%); more than half (59.3%) had no emotional exchange with their children.

**Table 4:** Basic characteristics of survey samples (*n* = 8,015).

**Variable**	**Category**	**Frequency**	**Percentage%**
Gender	Female	3900	48.7
	Male	4115	51.3
Age	60–69 years	5232	65.3
	70–79 years	2309	28.8
	Over 80 years	474	5.9
Marital status	Unmarried	1405	17.5
	Married	6610	82.5
Working status	Non-working	6770	84.5
	Working	1245	15.5
Education	Illiteracy	2019	25.2
	Primary school	3690	46
	Junior high school	1428	17.8
	Senior high school and above	878	11
Residence	Rural	5739	71.6
	Urban	2276	28.4
Type of insurance	No insurance	196	2.4
	Medical insurance for urban workers	1309	16.3
	Medical insurance for urban and rural residents	989	12.3
	Medical insurance for urban residents	350	4.4
	New rural cooperative medical insurance	4950	61.8
	Other	213	2.7
Chronic diseases	No	4252	53.1
	Yes	3763	46.9
Disabilities	No	6917	86.3
	Yes	1098	13.7
Intergenerational economic exchange pattern	No exchange	4326	54
	Only provide	514	6.4
	Only receive	2179	27.2
	Mutual support	996	12.4
Intergenerational care exchange pattern	No exchange	2280	28.4
	Only provide	745	9.3
	Only receive	3664	45.7
	Mutual support	1326	16.5
Intergenerational emotional exchange pattern	No exchange	4755	59.3
	Mutual support	3260	40.7
Intergenerational relationship quality	Extremely poor	90	1.1
	Not good	275	3.4
	Good	3130	39.1
	Very good	3935	49.1
	Excellent	585	7.3

### Bivariate correlations of the key variables

Table 5 shows the analysis of the correlation between various factors and depressive symptoms in the elderly. Results showed that in addition to age, other control variables, including gender, marital status, working status, education, residence, medical insurance status, chronic diseases, and disabilities, were significantly correlated with the depressive symptoms in the elderly; intergenerational economic, care and emotional exchange patterns had a significant impact on the presence or absence of depressive symptoms in the elderly; intergenerational relationship quality was significantly correlated with depressive symptoms in the elderly.

**Table 5:** Correlation analysis on the influencing factors of depressive symptoms (*n* = 8,015, *n*/%).

**Variable**	**Category**	**Non-depressive**	**Depressive**	***P*-value**
Gender	Female	2129 (54.59)	1771 (45.41)	0.000
	Male	2857 (69.43)	1258 (30.57)	
Age	60–69 years	3289 (62.86)	1943 (37.14)	0.132
	70–79 years	1397 (60.5)	912 (39.5)	
	Over 80 years	300 (63.29)	174 (36.71)	
Marital status	Unmarried	752 (53.52)	653 (46.48)	0.000
	Married	4234 (64.05)	2376 (35.95)	
Working status	Non-working	4081 (60.28)	2689 (39.72)	0.000
	Working	905 (72.69)	340 (27.31)	
Education	Illiteracy	1057 (52.35)	962 (47.65)	0.000
	Primary school	2215 (60.03)	1475 (39.97)	
	Junior high school	1025 (71.78)	403 (28.22)	
	Senior high school and above	689 (78.47)	189 (21.53)	
Residence	Rural	3358 (58.51)	2381 (41.49)	0.000
	Urban	1628 (71.53)	648 (28.47)	
Type of Insurance	No insurance	103 (52.55)	93 (47.45)	0.000
	Medical insurance for urban workers	1009 (77.08)	300 (22.92)	
	Medical insurance for urban and rural residents	625 (63.2)	364 (36.8)	
	Medical insurance for urban residents	239 (68.29)	111 (31.71)	
	New rural cooperative medical insurance	2844 (57.45)	2106 (42.55)	
	Other	161 (75.59)	52 (24.41)	
Chronic diseases	No	2883 (67.8)	1369 (32.2)	0.000
	Yes	2103 (55.89)	1660 (44.11)	
Disabilities	No	4478 (64.74)	2439 (35.26)	0.000
	Yes	508 (46.27)	590 (53.73)	
Intergenerational economic exchange pattern	No exchange	2774 (64.12)	1552 (35.88)	0.000
	Only provide	350 (68.09)	164 (31.91)	
	Only receive	1184 (54.34)	995 (45.66)	
	Mutual support	678 (68.07)	318 (31.93)	
Intergenerational care exchange pattern	No exchange	1305 (57.24)	975 (42.76)	0.000
	Only provide	430 (57.72)	315 (42.28)	
	Only receive	2395 (65.37)	1269 (34.63)	
	Mutual support	856 (64.56)	470 (35.44)	
Intergenerational emotional exchange pattern	No exchange	2839 (59.71)	1916 (40.29)	0.000
	Mutual support	2147 (65.86)	1113 (34.14)	
Intergenerational relationship quality	Extremely poor	18 (20)	72 (80)	0.000
	Not good	84 (30.55)	191 (69.45)	
	Good	1832 (58.53)	1298 (41.47)	
	Very good	2637 (67.01)	1298 (32.99)	
	Excellent	415 (70.94)	170 (29.06)	

### Regression analysis on influencing factors of the key variables

Table 6 shows three binary logistic regression models. Model 1 studied the effect of control variables on depressive symptoms in the elderly. Model 2, based on Model 1, included intergenerational exchange pattern variables in regression models to study the effect of intergenerational exchange patterns on depressive symptoms in the elderly after controlling control variables. Model 3, based on Model 2, included intergenerational relationship quality variables in regression models to study the effect of intergenerational relationship quality on depressive symptoms in the elderly after controlling control variables and intergenerational exchange pattern variables. Collinearity analysis was performed on three models before the inclusion of variables, and the results showed that VIF (Variance Inflation Factor) < 10 and no collinearity problems occurred (33). Meanwhile, the Hosmer test results showed *P* > 0.05, suggesting that the models fit well (34).

**Table 6:** Analysis of the relationship between intergenerational relationship quality and intergenerational exchange patterns and the depressive symptoms in the elderly (*n* = 8,015).

**Type of variable**	**Name of variable**	**Model 1**	**Model 2**	**Model 3**
Independent variables	Intergenerational relationship quality (extremely poor)			
	Not good			0.704
	Good			0.255***
	Very good			0.160***
	Excellent			0.144***
	Intergenerational economic exchange pattern (no exchange)			
	Only provide		0.878	0.863
	Only receive		1.050	1.088
	Mutual support		0.682***	0.719*
	Intergenerational care exchange pattern (no exchange)			
	Only provide		1.047	1.014
	Only receive		0.625**	0.674**
	Mutual support		0.616***	0.660***
	Intergenerational emotional exchange pattern (no exchange)			
	Mutual support		0.745***	0.759*
Control variables	Gender (male)			
	Female	1.597***	1.698***	1.728***
	Marital status (unmarried)			
	Married	0.773**	0.842*	0.898
	Working status (non-working)			
	Working	0.702**	0.738**	0.744***
	Education (illiteracy)			
	Primary school	0.967	0.964	0.945
	Junior high school	0.717**	0.701***	0.686***
	Senior high school and above	0.586***	0.571***	0.564***
	Residence (rural)			
	Urban	0.790*	0.788*	0.794*
	Type of insurance (no insurance)			
	Medical insurance for urban workers	0.512**	0.526**	0.503**
	Medical insurance for urban and rural residents	0.740	0.785	0.784
	Medical insurance for urban residents	0.679*	0.684	0.689
	New rural cooperative medical insurance	0.902	0.936	0.948
	Other	0.533*	0.542*	0.521*
	Chronic diseases (no)			
	Yes	1.632***	1.636***	1.619***
	Disabilities (no)			
	Yes	1.883***	1.876***	1.844***
	Constant	0.681*	0.927	4.047***
Mode fitting effect	Hosmer and lemeshow test	0.949	0.648	0.82

^*^p < 0.05; ^**^p < 0.01; ^***^p < 0.001.

Model 1 studied the effects of 8 control variables, including gender, marital status, working status, education, residence, medical insurance status, chronic diseases, and disabilities, on depressive symptoms of the elderly. Among them, gender is a risk factor for depressive symptoms; the risk of depressive symptoms in older women was significantly higher (1.597 times) than in older men. Being working and married are protective factors for depressive symptoms in the elderly; the risk of depressive symptoms in the working elderly was 29.8% lower than in non-working ones, and that in the married was 22.7% lower than the unmarried. With the improvement of educational levels, the elderly's depressive symptoms were somewhat reduced. Compared with illiterate older people, there was no significant difference in the risk of depressive symptoms in older people with primary school education, while there were significant differences in those with junior high school education and those with senior high school education and above (the risk of depressive symptoms decreased by 28.3 and 41.4%, respectively). Compared with the elderly without medical insurance, there was no significant difference in the risk of depressive symptoms in those with new rural cooperative medical insurance and medical insurance for urban and rural residents, while there were significant differences in those with basic medical insurance for urban workers, medical insurance for urban residents, and other medical insurance (the risk of depressive symptoms decreased by 48.8, 31.2, and 46.7%). Diseases are a risk factor for depressive symptoms in the elderly; older people with chronic diseases and disabilities had a significantly increased risk of depressive symptoms, which was 1.632 and 1.883 times that of healthy people.

After controlling control variables, Model 2 studied the effects of different intergenerational exchange patterns on the risk of depressive symptoms in the elderly. In the economic exchange pattern, compared with the group having no exchange, there were significant differences in the risk of depressive symptoms in the elderly in the mutual support group (the risk in the mutual support group was 31.8% lower than the group having no exchange), while there was no significant difference in the group only providing support and the group receiving support. In the care exchange pattern, compared with the group having no exchange, there were significant differences in the risk of depressive symptoms in the elderly in the group receiving support and the mutual support group (the risks in the two groups were 37.5 and 38.4% lower than the group having no exchange, respectively), while there was no significant difference in the group only providing support. In the emotional exchange pattern, there were significant differences in the risk of depressive symptoms in the elderly between the group having no support and the mutual support group (the risk in the latter was 25.5% lower than in the former). To sum up, the risks of depressive symptoms in the elderly in the mutual support group were significantly lower than those in the group having no exchange in the economic, care, and emotional exchange patterns.

**Figure 1:**
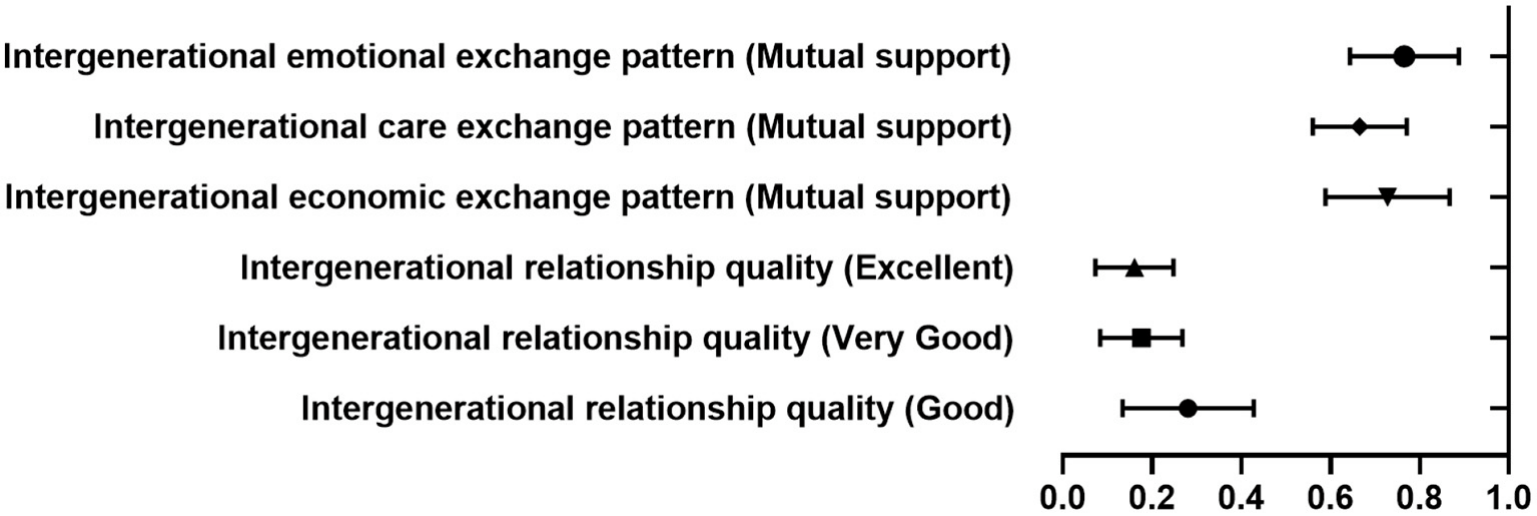
Forest diagram of factors influencing depressive symptoms in the elderly.

Model 3 studied the effect of intergenerational relationship quality on depressive symptoms in the elderly after optimizing control variables and intergenerational exchange pattern variables. There were significant differences in the risk of depressive symptoms in the elderly with extremely poor intergenerational relationships and other groups, and the risks in the elderly with good, very good, and excellent intergenerational relationships were 74.5, 84.0, and 85.6% lower than those with extremely poor intergenerational relationships. The results are shown in Figure 1. This shows that intergenerational relationship quality is a protective factor toward depressive symptoms in the elderly, and the risk of depressive symptoms in the elderly gradually decreases as intergenerational relationship quality improves.

## Discussion

### Effects of the intergenerational exchange pattern on depressive symptoms in the elderly

In different exchange patterns, the elderly have the lowest risk of depressive symptoms when there is mutual economic, care, and emotional support between generations, which is consistent with several conclusions based on Chinese data. For example, Dura et al. (35) found that intergenerational exchange with children could directly enhance parents' well-being; Huang et al. (26) found that intergenerational exchange was conducive to the elderly's self-rated health, and the closer the intergenerational exchanges, the better for the elderly's physical and mental health. Nevertheless, most studies based on non-Chinese data have not supported this conclusion. Lowenstein et al. (26) found that older people were more likely to be the “givers” in intergenerational exchange, in which they could realize their self-value while receiving support from their children could only hurt their self-esteem and thus affect their mental health. Cultural differences may be the root cause of the differences in research results. In western countries where freedom and independence are advocated, parents are responsible for bringing up their children, but children have no obligation to support their parents (36). In this case, parents play the role of a “giver.” On the contrary, in China and even East Asia, the norm of filial piety still significantly affects family relations. Exchange is sustainable only through reciprocity and balance between generations (37). We can see from family relationships that the elderly feel depressed when they are unable to support their children, thus hurting their mental health. They perceive themselves as responding to their children's support and recognize that they are needed. This move can improve their self-esteem and thus reduce the risk of depression (24). This is in line with the results of this study, which showed that the mutually supportive intergenerational exchange model could greatly reduce the risk of depression in the elderly.

### Effects of intergenerational relationship quality on depressive symptoms in the elderly

As the intergenerational relationship quality enhances, the risk of depressive symptoms in the elderly significantly decreases. Compared with the results obtained using the intensity of intergenerational exchange to expect intergenerational relationship quality, this study directly measured intergenerational relationship quality with the elderly's subjective evaluation, which was more significantly correlated with the level of depressive symptoms in the elderly (27, 28). The reason may be that the high intensity of intergenerational exchange does not inevitably mean good intergenerational relationships, and negative intergenerational exchange (such as quarrels and frequently asking for money) may cause trouble for older people and thus increase the risk of depressive symptoms. The quality of intergenerational relationships is a cross-cultural concept, and good intergenerational relationships are an important determinant of older adults' well-being wherever and whenever they occur (27, 38). In China, home-based care, as the most traditional form of old-age care, still has a major position (39), while the quality of the intergenerational relationship, the core social relationship in the elderly's life, has a greater impact on the elderly. According to Amato and Booth, intergenerational support should be influenced not only by demographics but also by prospects for intergenerational interaction such as the number and availability of relatives (40). This means that the study of intergenerational support should be performed not only at the behavioral level, such as frequency of contact, giving, and receiving of support, but also at the emotional level, such as the feelings, values, and perspectives of intimacy and connection. In addition, intergenerational support is affected not only by the measurable exchange but also by normative issues such as the amount of support given and received between members, the importance of filial duty, and values in the parent-child relationship (41). This study suggests that improving the quality of intergenerational relationships can effectively reduce the risk of depressive symptoms in the elderly.

### Effects of multiple factors on depression in the elderly

The depressive symptoms in the studied elderly were high, with significant internal heterogeneity. According to the WHO report in 2017, the prevalence of depressive symptoms was 7.5% in women and over 5.5% in men aged 55–74 years (42). In the sample in this paper, up to 37.80% of the elderly had depressive symptoms, including 30.57% of men and 45.41% of women, much higher than the world average, suggesting that the depressive symptoms in the elderly are serious in China. There were significant differences in the degree of depressive symptoms in the elderly of different genders, marriage, urban and rural areas, education level, type of insurance, and health status. Compared with the urban elderly, the rural elderly suffered from a significantly higher risk of depression. Perhaps, due to the impact of urbanization and population aging, young people in rural areas flooding into cities for better development, and the increase of empty-nested elderly, these factors exacerbate the aging degree in rural areas. The elderly living alone have difficulties communicating with their children without intergenerational emotional support and intergenerational care support, thus creating an unrelieved depression in the elderly in rural areas. In parallel, studies have shown that rural areas are provided with scarce health care resources and low availability of medical professionals (43). These negative factors lead to deeper depression in the elderly who cannot get help in time.

Under the background of increasing aging, it is necessary to improve the quality of intergenerational relationships and establish a positive intergenerational exchange mode to reduce the level of depression in the elderly. Firstly, the government should encourage intergenerational communication by providing more adequate family leave and more time for intergenerational communication. Secondly, society should provide daily care services for the elderly, including routine physical examination, psychological consultation, and daily activity places, to meet the needs of the elderly in time. Thirdly, family members should take the initiative to communicate with the elderly; children should actively meet or call them, and teachers should learn to use communication tools to help the elderly keep up with the pace of society as much as possible. Lastly, the elderly should also learn to change their mindset, seek help from their children, and strengthen mutual support between generations.

## Limitation

There are several limitations to this study. First, intergenerational relationship quality is a dimensionally rich concept, and it is inadequate to measure it using a single subjective indicator. Emotional evaluation is somewhat subjective and unstable; therefore, the results should be interpreted more carefully. Second, the causal relationship should also be determined carefully in cross-sectional studies, and if conditions permit, targeted follow-up can be carried out to verify the conclusions of this study. In the future, the panel data can be used to further verify the conclusions of this paper. Alternatively, targeted follow-up surveys can be performed focusing on the measurement indicators of intergenerational relationship quality to further explore the relationship between intergenerational relationship quality and depression in the elderly. Additionally, we look forward to continuing exploring the gender and urban-rural differences in the influence of intergenerational relationships on depression in the elderly to reduce their level of depression and provide more targeted suggestions for active aging.

## Conclusion

In conclusion, intergenerational relationship quality and intergenerational exchange patterns significantly affect depressive symptoms in the elderly, and high-quality intergenerational relationships and the intergenerational exchange pattern of mutual support can greatly reduce the risk of depressive symptoms in the elderly. This study not only added to the theories of related studies but also pointed in the right direction for reducing the severity of depressive symptoms in the elderly. The quality of intergenerational relationships can be improved by encouraging closer economic, care, and mutual emotional support between generations.

## Data availability statement

Publicly available datasets were analyzed in this study. This data can be found here: https://charls.pku.edu.cn.

## Ethics statement

All the respondents signed informed consent at the time of participation, and this study was approved by the Institutional Review Board of Peking University (IRB00001052-11014).

## Author contributions

RZ took responsibility for the integrity of the data and the accuracy of the data analysis. RZ, MY, and GL study design. JZ, LH, BG, FW, and DF contributed to the writing of the manuscript and statistical analysis. JZ and GL study supervision. All authors contributed to this article and approved the submitted version.

## Conflict of interest

The authors declare that the research was conducted without any commercial or financial relationships that could be construed as a potential conflict of interest.

## Publisher's note

All claims expressed in this article are solely those of the authors and do not necessarily represent those of their affiliated organizations, or those of the publisher, the editors and the reviewers. Any product that may be evaluated in this article, or claim that may be made by its manufacturer, is not guaranteed or endorsed by the publisher.

## References

[1] Li N Chen G Zeng P Pang J Gong H Han Y. Prevalence of depression and its associated factors among Chinese elderly people: a comparison study between community-based population and hospitalized population. Psychiatry Res. (2016) 243:87–91. 10.1016/j.psychres.2016.05.030 27376667

[2] Chollet F Cramer SC Stinear C Kappelle LJ Baron JC Weiller C. Pharmacological therapies in post stroke recovery: recommendations for future clinical trials. J Neurol. (2014) 261:1461–8. 10.1007/s00415-013-7172-z 24221642

[3] Jorn C Ute L. Adapted psychotherapy for suicidal geriatric patients with depression. BMC Psychiatry. (2018) 18:203. 10.1186/s12888-018-1775-y 29914407 PMC6006781

[4] Qin BY Dai LL Zheng Y. Efficacy of repetitive transcranial magnetic stimulation for alleviating clinical symptoms and suicidal ideation in elderly depressive patients: a randomized controlled trial. Nan Fang Yi Ke Da Xue Xue Bao. (2017) 37:97–101. 10.3969/j.issn.1673-4254.2017.01.18 28109107 PMC6765751

[5] Chan ASW Lo IPY Yan E. Health and social inclusion: the impact of psychological well-being and suicide attempts among older men who have sex with men. Am J Men's Health. (2022) 16:15579883221120985. 10.1177/15579883221120985 36082415 PMC9465597

[6] National Bureau of Statistics of People's Republic of China. China Population Census Yearbook. Beijing: China Statistics Press (2020).

[7] World Health Statistics. Monitoring Health for the SDGs. Boston, MA: World Health Statistics (2018).

[8] Chan ASW Ho JMC Tam HL Hsu WL Tang PMK. COVID-19, SARS, and MERS: the risk factor associated with depression and its impact on psychological well-being among sexual moralities. J Psychiatry Behav Sci. (2022) 5:1073.

[9] Chan A Ho J Li J Tam HL Tang P. Impacts of COVID-19 pandemic on psychological well-being of older chronic kidney disease patients. Front Med. (2021) 8:666973. 10.3389/fmed.2021.666973 34124096 PMC8187602

[10] Chan ASW Ho JMC Tam HL Tang PMK. Book review: successful aging: a neuroscientist explores the power and potential of our lives. Front Psychol. (2021) 2234:705368. 10.3389/fpsyg.2021.705368

[11] Ho JMC Chan ASW Luk CY Tang PMK. Book review: the body keeps the score: brain, mind, and body in the healing of trauma. Front Psychol. (2021) 2021:2383. 10.3389/fpsyg.2021.704974

[12] Schwarzbach M Luppa M Forstmeier S König HH Riedel-Heller SG. Social relations and depression in late life-a systematic review. Int J Geriatr Psychiatry. (2014) 29:1–21. 10.1002/gps.3971 23720299

[13] Gariépy G Honkaniemi H Quesnel-Vallée A. Social support and protection from depression: a systematic review of current findings in Western countries. Br J Psychiatry J Mental Sci. (2016) 209:284–93. 10.1192/bjp.bp.115.169094 27445355

[14] Werner-Seidler A Afzali MH Chapman C Sunderland M Slade T. The relationship between social support networks and depression in the 2007 national survey of mental health and well-being. Soc Psychiatry Psychiatr Epidemiol. (2017) 52:1463–73. 10.1007/s00127-017-1440-7 28889230

[15] Marie YS Montgomery RJ Kosloski K. A dimensional analysis of caregiver burden among spouses and adult children. Gerontologist. (2011) 51:321–31. 10.1093/geront/gnq102 21135026

[16] Fingerman KL Sechrist J Birditt K. Changing views on intergenerational ties. Gerontology. (2013) 59:64–70. 10.1159/000342211 23037718 PMC4480642

[17] Tang S Yang T Ye C Liu M Gong Y Yao L. Research on grandchild care and depression of Chinese older adults based on CHARLS2018: the mediating role of intergenerational support from children. BMC Public Health. (2022) 22:137. 10.1186/s12889-022-12553-x 35045856 PMC8772115

[18] Åhlin J Hallgren M Öjehagen A Källmén H Forsell Y. Adults with mild to moderate depression exhibit more alcohol related problems compared to the general adult population: a cross sectional study. BMC Public Health. (2015) 15:542. 10.1186/s12889-015-1837-8 26051511 PMC4459061

[19] Van de Velde S Bracke P Levecque K. Gender differences in depression in 23 European countries. Cross-national variation in the gender gap in depression. Soc Sci Med. (2010) 71:305–13. 10.1016/j.socscimed.2010.03.035 20483518

[20] Roh HW Lee Y Lee KS Chang KJ Kim J Lee SJ. Frequency of contact with non-cohabitating adult children and risk of depression in elderly: a community-based 3-year longitudinal study in Korea. Arch Gerontol Geriatr. (2015) 60:183–9. 10.1016/j.archger.2014.09.007 25442783

[21] Teixeira AR Wender MH Gonçalves AK Freitas C Santos AM Soldera CL. Dizziness, physical exercise, falls, and depression in adults and the elderly. Int Arch Otorhinolaryngol. (2016) 20:124–31. 10.1055/s-0035-1566304 27096016 PMC4835334

[22] Bonsang E Bordone V. The effect of informal care from children on cognitive functioning of older parents. Develop Utilisat Hum Resour Semin Ser. (2013) 8:1–35. 10.2139/ssrn.2251784

[23] He G Xie JF Zhou J-D Zhong Z-Q Qin C-X Ding S-Q. Depression in left-behind elderly in rural China: prevalence and associated factors. Geriatr Gerontol Int. (2016) 16:638–43. 10.1111/ggi.12518 26017357

[24] Abolfathi Momtaz Y Ibrahim R Hamid TA. The impact of giving support to others on older adults' perceived health status. Psychogeriat Off J Jpn Psychogeriat Soc. (2014) 14:31–7. 10.1111/psyg.12036 24299124

[25] Xiong B Shi R. How intergenerational relationship influences intergenerational support in Chinese family: based on the perspective of the elderly parents. Populat J. (2016) 5:102–11. 10.16405/j.cnki.1004-129X.2016.05.011

[26] Huang Q Hu Y Chen G. Effects of intergenerational support on health among elderly: a study based on the perspective of social exchange theory. Populat Develop. (2017) 23:43–5. 10.3969/j.issn.1674-1668.2017.01.005

[27] Yang J Ariela L Todd J Yong Z. Intergenerational latent solidarity class and relationship quality among Chinese: implications for self-reported health and well-being. Acta Psychol Sin. (2013) 45:811–24. 10.3724/SP.J.1041.2013.00811

[28] Andresen EM Malmgren JA Carter WB Patrick DL. Screening for depression in well older adults: evaluation of a short form of the CES-D. Am J Prev Med. (1994) 10:77–84. 10.1016/S0749-3797(18)30622-6 8037935

[29] Liu Y-G Wang C-C Huang Q Zhang L Liu Y. Association of vision and hearing status with depressive symptoms among middle-aged and older Chinese adults [Original Research]. Front Public Health. (2022) 10:857307. 10.3389/fpubh.2022.857307 35979465 PMC9376298

[30] Qingbo H Xiaohua W Gong C. Reliability and validity of 10-item CES-D among middle aged and older adults in China. China J Health Psychol. (2015) 7:1036–41. 10.13342/j.cnki.cjhp.2015.07.023

[31] Köhler CA Evangelou E Stubbs B Solmi M Veronese N Belbasis L. Mapping risk factors for depression across the lifespan: an umbrella review of evidence from meta-analyses and Mendelian randomization studies. J Psychiatr Res. (2018) 103:189–207. 10.1016/j.jpsychires.2018.05.020 29886003

[32] Fernandez-Rodrigues V Sanchez-Carro Y Lagunas LN Rico-Uribe LA Pemau A Diaz-Carracedo P. Risk factors for suicidal behavior in late-life depression: a systematic review. World J Psychiatry. (2022) 12:187–203. v12.i1.187. 10.5498/wjp.v12.i1.187 35111588 PMC8783161

[33] O'Brien RM. A caution regarding rules of thumb for variance inflation factors. Qual Quant. (2007) 41:673–90. 10.1007/s11135-006-9018-6

[34] Hosmer DW Hosmer T Le Cessie S Lemeshow S. A comparison of goodness-of-fit tests for the logistic regression model. Stat Med. (1997) 16:965–80. 10.1002/(SICI)1097-0258(19970515)16:9&lt;965::AID-SIM509&gt;3.0.CO;2-O 9160492

[35] Li C Jiang S Zhang X. Intergenerational relationship, family social support, and depression among Chinese elderly: a structural equation modeling analysis. J Affect Disord. (2019) 248:73–80. 10.1016/j.jad.2019.01.032 30716614

[36] Lowenstein A Katz R Gur-Yaish N. Reciprocity in parent-child exchange and life satisfaction among the elderly: a cross-national perspective. J Soc Issues. (2007) 63:865–83. 10.1111/j.1540-4560.2007.00541.x

[37] Antonucci TC Ajrouch KJ Manalel JA. Social relations and technology: continuity, context, and change. Innovat Aging. (2017) 1:igx029. 10.1093/geroni/igx029 29795794 PMC5954608

[38] Antonucci TC Ajrouch KJ Birditt KS. The convoy model: explaining social relations from a multidisciplinary perspective. Gerontologist. (2014) 54:82–92. 10.1093/geront/gnt118 24142914 PMC3894851

[39] Huang H Zhang L. Present situation and thinking of various pension modes and service technologies. Chin J Gerontol. (2021) 41:203–7. 10.3969/j.issn.1005-9202.2021.01.057

[40] Amato P Booth A. A generation at risk: growing up in an era of family upheaval. By Paul R Amato Alan Booth Soc Forces. (1999) 78:396–7. 10.2307/3005818

[41] Szinovacz B editor. Handbook on Grandparenthood. Westport, CT: Greenwood Press (1998).

[42] World Health Statistics. Depression and Other Common Mental Disorders, Global Health Estimates. Boston, MA: World Health Statistics (2017).

[43] Tam HL Chung SF Wang Q. Urban-rural disparities in hypertension management among middle-aged and older patients: results of a 2018 Chinese national study. Chronic Illn. (2022) 2022:17423953221102627. 10.1177/17423953221102627 35603631

